# Light exposure mediates circadian rhythms of rhizosphere microbial communities

**DOI:** 10.1038/s41396-021-00957-3

**Published:** 2021-03-21

**Authors:** Kankan Zhao, Bin Ma, Yan Xu, Erinne Stirling, Jianming Xu

**Affiliations:** 1 Institute of Soil and Water Resources and Environmental Science, College of Environmental and Resource Sciences, Zhejiang University, Hangzhou, China; 2 Zhejiang Provincial Key Laboratory of Agricultural Resources and Environment, Zhejiang University, Hangzhou, China; 3 Hangzhou Global Scientific and Technological Innovation Center, Zhejiang University, Hangzhou, China; 4 Acid Sulfate Soils Centre, School of Biological Sciences, The University of Adelaide, Adelaide, SA Australia

**Keywords:** Microbial ecology, Community ecology

## Abstract

Microbial community circadian rhythms have a broad influence on host health and even though light-induced environmental fluctuations could regulate microbial communities, the contribution of light to the circadian rhythms of rhizosphere microbial communities has received little attention. To address this gap, we monitored diel changes in the microbial communities in rice (*Oryza sativa* L.) rhizosphere soil under light–dark and constant dark regimes, identifying microbes with circadian rhythms caused by light exposure and microbial circadian clocks, respectively. While rhizosphere microbial communities displayed circadian rhythms under light–dark and constant dark regimes, taxa possessing circadian rhythms under the two conditions were dissimilar. Light exposure concealed microbial circadian clocks as a regulatory driver, leading to fewer ecological niches in light versus dark communities. These findings disentangle regulation mechanisms for circadian rhythms in the rice rhizosphere microbial communities and highlight the role of light-induced regulation of rhizosphere microbial communities.

## Introduction

The Earth’s rotation, and thus daily cycles of light and dark, has influenced living organisms throughout evolutionary time [1]. As a result, most organisms have an inherent ability to measure the passage of time at a circa 24-h scale by using daily biological oscillations called circadian clocks [2]. Circadian clocks synchronize an organism’s behavioral and physiological processes to periodic environmental factors and anticipate future rhythmic environmental changes [3]. These rhythms allow temporal mutualism and reduce competition among sympatric species, thereby promoting the survival of organisms in rhythmic environments [4].

The short reproductive time (<24 h) of most prokaryotes has been used as evidence that these organisms might not possess circadian clocks [4]. Even so, various prokaryotes have been observed with circadian growth patterns on agar plates and in complex natural ecosystems [5, 6], suggesting the existence of prokaryotic circadian clocks. Cyanobacteria were the first prokaryotes reported to have a core circadian oscillator consisting of *KaiA, KaiB,* and *KaiC* [7]. Evolutionarily, *KaiC* and its homologs are the oldest oscillators among the three [8, 9], which have been found in Proteobacteria, Thermotogae, Chloroflexi and Euryarchaeota [10–13]. Furthermore, oscillations in peroxiredoxin proteins observed in model organisms suggest that these proteins constitute a universal and conserved marker for circadian rhythms [14].

Light regulates numerous physiological processes in plants [15]. It influences enzyme expression in many plant pathways, including chlorophyll biosynthesis, electron transport photosystems, starch synthesis and degradation, and nitrogen/sulfur assimilation [16]. Plant carbon partitioning and root-soil nutrient exchange are also controlled by light [17, 18]. Plant responses to diurnal changes in light naturally lead to rhizosphere diurnal fluctuations in soil pH, oxygen content, nutrient contents, and antimicrobial compounds due to the diel consumption/production of resources and waste materials [19–21].

Light exposure is an upstream factor that governs the circadian rhythms of host microbiomes in vivo [22, 23]. Gut microbial communities exhibit circadian rhythms in response to the time of day under normal light conditions [24–27] and eventually lose their circadian rhythms when the host is exposed to constant light or dark conditions. These dynamics indicate complex relationships between host exposure to diurnal variation and microbial circadian rhythms [22, 28]. Similarly, rhizosphere microbial communities show circadian rhythms under cyclic light–dark (LD) conditions [29, 30]. Although several microbial taxa display diurnal fluctuations in abundance, it is not clear if this is a response to changes in the rhizosphere environment caused by root activities or microbial endogenous circadian clocks [30].

The molecular circadian clock is a conserved attribute that is protected from environmental changes within physiologically permissible limits [2]. In order to identify prokaryotic taxa with circadian clocks, we used the most common indicator: a circa 24-h oscillation of microbial abundance under otherwise constant environmental conditions [4]. Rhizosphere soil samples were collected at the stem elongation growth stage of rice grown under a 24-h LD cycle for 3 days or under constant dark (DD) conditions for 72 h to identify microorganisms with circadian rhythms in LD or DD regimes. Our results indicate that rhizosphere microbial communities differ between day and night in each treatment, and show that circadian microorganisms perform differently under altered light conditions. These findings expand our knowledge of the adaptive capacity of circadian rhythms and provide a foundation for controlling rhizosphere microbial community structure and function through light exposure.

## Materials and methods

### Experimental materials and rice cultivation

Experimental soil was collected from a rice field in Jiaxing, China (30° 50′ 8.74″ N, 120° 43′ 3.68″ E); basic physicochemical properties have been previously described [31]. For our experiment, soil was sieved with a 2-mm mesh and used as a media in plastic pots (diameter = 57 mm, height = 155 mm, 200 g dry soil per pot). Seeds of *Oryza sativa* L. cultivar Yongyou 12 were surface-sterilized, germinated and cultivated for 2 weeks as previously reported [31], after which uniform seedlings were selected and transplanted into the potted soil at a density of one plant per pot for a total of 84 pots (2 treatments × 6 sampling times × 7 replicates). Unplanted soil was used as a negative control for an additional 30 pots (6 sampling times × 5 replicates). All pots were kept flooded and under greenhouse conditions; greenhouse conditions were 12 h light (9 am to 9 pm) and 12 h dark (9 pm to 9 am) with constant temperature and relative humidity (28 °C; 70%).

### Experimental design and sampling

After 60 days of growth, plants were divided into two groups: one was given the regular LD cycle as described above and the other received a DD environment for 3 days. For both groups, seven replicates were harvested every 12 h at 8 am (AM) and 8 pm (PM); harvest occurred 1 h prior to the light condition change in the LD group. Rhizosphere soil was defined as soil tightly adhering to the roots (*n* = 7) and while bulk soil was collected from unplanted soil (*n* = 5) [32, 33]; plant matter was excluded from the rhizosphere soil. Soil was collected in an RNase-free tube with 3 mL LifeGuard Soil Preservation Solution (MoBio Laboratories, Carlsbad, CA, USA) per gram of sample. Samples were homogenized by hand-mixing, then immediately frozen with liquid nitrogen and stored at −80 °C until RNA extraction.

During the 72-h sampling period, in situ rhizosphere oxygen and pH profiles were determined using oxygen and pH microelectrode sensors (Unisense OXY25 and pH-N; Science Park, Aarhus, Denmark). The microsensor tip was inserted into the soil adhering to the roots and measured every 90 s. For each harvest time point, rhizosphere soil was sampled for dissolved organic carbon (DOC) concentration using a 1:5, soil:water extraction and quantification by TOC analyzer (Multi N/C 3100, Analytik Jena AG, Jena, Germany).

### RNA extraction and 16S cDNA amplicon sequencing

Soil total RNA was extracted with E.Z.N.A. Soil RNA Mini Kits and purified with E.Z.N.A. RNase-Free DNase I Set according to the manufacturer’s protocols (Omega Bio-tek, Norcross, GA, USA). After extraction, RNA was used in reverse transcription to generate complimentary DNA (cDNA) by PrimeScript II 1st strand cDNA Synthesis Kits (Takara, Dalian, China). 16S cDNA was amplified by primers 515F (5′-GTGCCAGCMGCCGCGGTAA-3′) and 907R (5′-CCGTCAATTCCTTTGAGTTT-3′) [34]. PCR conditions were as follows: 98 °C for 1 min; 30 cycles at 98 °C (10 s), 50 °C (30 s) and 72 °C (30 s); and 72 °C for 5 min. Amplified PCR products were sequenced on an Illumina HiSeq PE250 sequencing platform (Illumina, San Diego, CA, USA). Sequences were clustered into operational taxonomic units (OTUs) with VSEARCH-2.11.1 [35] with a sequence similarity threshold of 0.97. Ribosomal database project training set v16 [36] was used for taxonomy annotations at a threshold of 0.8 using the SINTAX algorithm [37]. All sequence data were deposited into Genome Sequence Archive under PRJCA003001 and PRJCA003009.

### Determination of 16S cDNA gene copies by real-time quantitative PCR

To calculate the absolute abundance of prokaryotes, qPCR was performed on the cDNA samples to determine the copy number of 16S cDNA [38] by LightCycler 480II (Roche, Mannheim, Germany). Concentration of cDNA was determined by a Nanodrop ND-2000 spectrophotometer (NanoDrop Technologies, Wilmington, DE, USA). PCR primers were chosen as above and PCR conditions were as follows: 95 °C for 2 min; 40 cycles at 95 °C (1 min), 49 °C (30 s) and 72 °C (50 s); and 72 °C for 5 min. The qPCR reaction mixture profiles and data analysis methods were conducted as before [39]. Amplification efficiencies of standard curves were 91–97% with *R*^2^ values of 0.992–0.999.

### Microbial community analysis

Microbial community analysis used absolute abundance and was conducted using R (version 3.6.1; R Core Team, Vienna, Austria). The community matrix was normalized using R package *DESeq* [40]. Principal coordinates analysis of Weighted UniFrac distance [41] was chosen to show microbial community variations between samples. Genera with statistically significant differences in normalized abundance between day and night, that also displayed the same change trends between day and night were regarded as taxa with circadian rhythms. That is, if one genus is significantly lower in the evening, it must be lower than its adjacent time points in order to be considered a ‘circadian taxon’. A random forests approach [42] identified the top 30 (rhizosphere) and top 40 (bulk soil) important genera responsible for day–night differences in the two experimental groups using R package *randomForest* [43, 44]. To show the interactions among species in different communities, microbial co-occurrence networks were constructed has been previously described [45]. Network Enhancement [46] was used to denoise undirected weighted biological networks using the R package *neten*. The cutoff of correlation coefficients was determined through random matrix theory-based methods [47] using the R package *﻿RMThreshold*. We generated sub-networks from four meta-community networks by preserving genera presented at each time point by the *subgraph* function in the R package *igraph* [48]. To compare our networks against random effects on network generation, we also generated random networks with same node number for each time point using the *erdos.renyi.game* function in *igraph* [48]. Network plots were visualized using *Gephi* [49] and topological features for each network were calculated using *igraph* [48].

### Statistical analysis

We assessed significant differences for 16S cDNA copy number at the community level and genus level and α-diversity across different groups using one-way analysis of variance (ANOVA) and Tukey’s Honest Significant Difference (HSD). Permutational multivariate analysis of variance (PERMANOVA) was employed to evaluate significant differences between groups by the R package *Vegan* [50].

## Results

### Rhythms in rice rhizosphere physicochemical properties

**Fig. 1:**
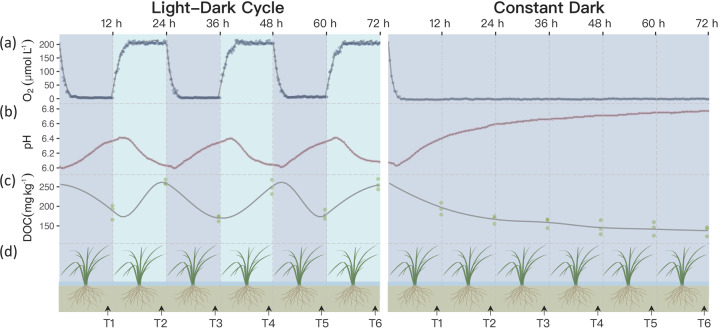
Diurnal changes in rice rhizosphere environment for the light-dark cycle and constant dark treatments. **a** Oxygen content. **b** Rhizosphere pH. **c** Dissolved organic carbon concentration. **d** Experimental design.

Rhizosphere physicochemical properties displayed regular rhythms under LD cycles. Oxygen concentration increased to ~200 μmol L^−1^ after 3 h under light conditions and decreased to ~5 μmol L^−1^ in 3 h under dark conditions (Fig. 1a). Soil pH decreased from 6.4 to 6.0 during the day and recovered to 6.4 during the night; there was a circa 1-h delay in pH response to the LD cycle. DOC concentration was significantly higher (Tukey HSD, *n* = 21, *P* < 0.001) during the day (231–270 mg kg^−1^ soil) than at night (161–201 mg kg^−1^ soil, Fig. 1c). The oxygen, pH and DOC rhythms were not present in the DD treatment; DD soils maintained a constant oxygen concentration of ~5 μmol L^−1^, a slow increase of pH to 6.8, and a continuous decrease in DOC (to ~140 mg kg^−1^ soil) over the 72-h experimental period.

### Circadian rhythms in the microbial communities in rice rhizosphere and bulk soils

**Fig. 2:**
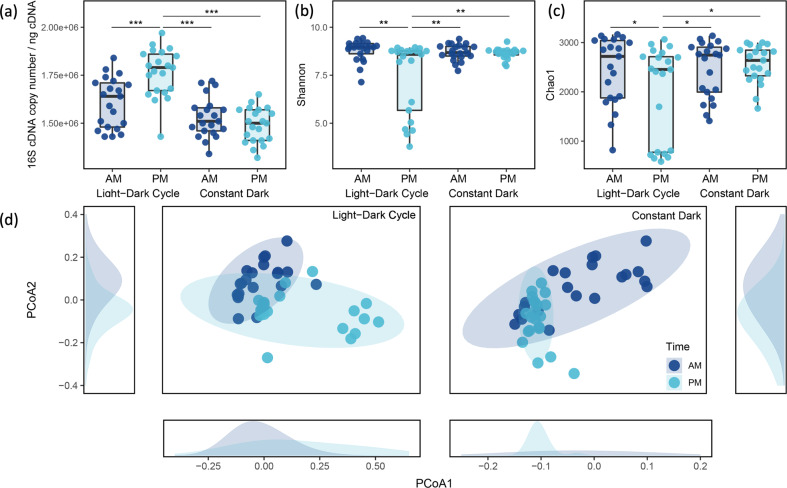
Circadian rhythms in the rhizosphere microbial communities. **a** 16S cDNA copy number in each sample. **b** Alpha diversity based on Shannon index in all four rhizosphere groups (*n* = 21 for each group). Boxes are vertically bounded by the 1st and 3rd quartiles, center line is median, and whiskers extend to ≤1.5x inter-quantile-range. **c** Alpha diversity based on Chao1 index. **d** Principal coordinate analysis (PCoA) of weighted unifrac dissimilarities (*n* = 21 for each group). Curvilinear polygons show estimations of frequency densities. PCoA ellipses indicate 95% confident interval.

Microbial activity in rice rhizosphere soil, as quantified by the copy number of 16S rRNA genes using metatranscriptomics, fluctuated with the LD cycle (Fig. 2a and Fig. S1). Microbial activity was significantly higher by day than night in the LD regime (Tukey HSD, *n* = 21, *P* < 0.001), a response not observed in the DD regime. Rhizosphere microbial activity was significantly lower in the DD samples than the LD samples (Tukey HSD, *n* = 42, *P* < 0.001).

Light exposure significantly decreased α-diversity of the rice rhizosphere microbial communities (Fig. 2b, c). The α-diversity was significantly lower in PM (end of light period) versus AM measurements of LD samples (Tukey HSD, *n* = 21, *P* = 0.005 for Shannon index and *P* = 0.017 for Chao1 index), but did not fluctuate in the DD regime (Tukey HSD, *n* = 21, *P* > 0.05 for both Shannon and Chao1 indices). Rhizosphere α-diversity during dark measurements was similar for both LD and DD samples (Tukey HSD, *n* = 21, pairwise *P* > 0.05 for both Shannon and Chao1 indices).

Rhizosphere microbial communities were significantly different between day and night under both LD and DD treatments (Fig. 2d, Fig. S2). The turnover rate of rhizosphere microbial communities was significantly higher under LD than DD (Supplementary Table S1). Night measurements were not statistically different between LD than DD treatments.

To differentiate plant-induced influences from internal microbiota changes, bulk soil was also collected. Compared to rhizosphere communities, bulk soil communities had a higher α-diversity but lower activity (Fig. S3a–c). However, similar to rhizosphere communities in the DD regime, bulk soil communities also had distinct community structures between day and night in both LD and DD treatments (Fig. S3d, Supplementary Table S2) even though there were no significant differences in community composition or activity between the four sampling conditions (i.e., DD, LD, AM, and PM).

### Circadian rhythms in rhizosphere and bulk soil microbial genera

**Fig. 3:**
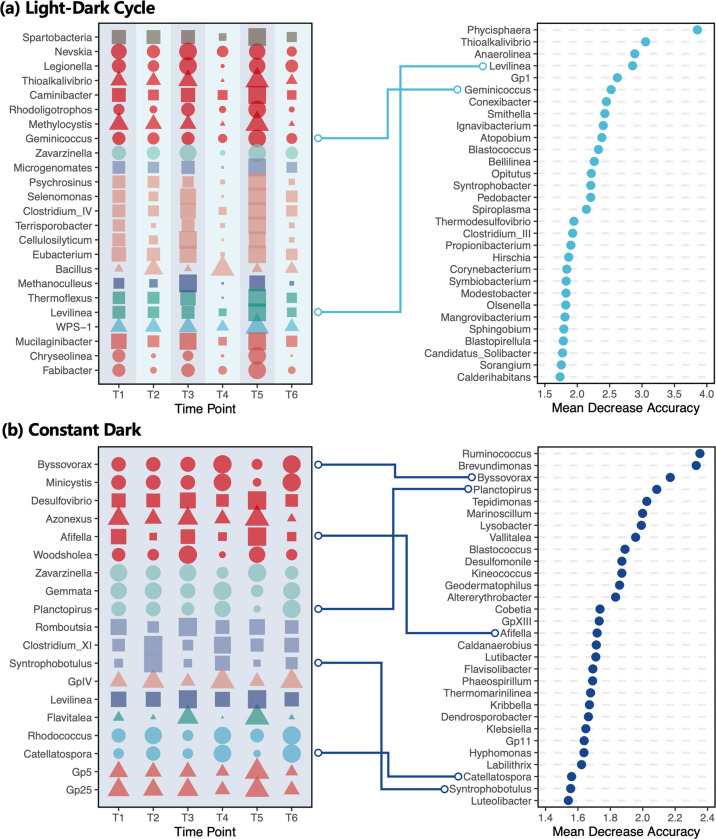
Rhizosphere microbiota with identified circadian rhythms (left) and the top 30 indicator taxa associated with AM and PM measurements (right). **a** Light-dark cycle treatment. **b** Constant dark treatment. Within the left panel, bubble size indicates normalized abundance of a genus along six time points wherein the population of each genus at each time point was compared to the maximum population of each genera within all time points. Key to bubble shape: square, obligate anaerobic taxa; triangle, anaerobic, or anaerobic taxa; circle, obligate aerobic taxa. Bubble colors represent phyla. Polylines connect genus that are identified as both circadian taxa and indicator taxa.

Within the rhizosphere communities, the proportion and classification of taxa with circadian rhythms differed between the LD and DD treatments: ~12.2% of the microbial communities (24 genera) showed daily variation in the LD group, while only ~7.4% (19 genera) responded similarly in the DD group (Fig. 3). Of the genera with identified circadian rhythms, only *Levilinea* was shared by both groups. Within the bulk soil communities, however, the taxa with circadian rhythms were much more similar between the LD and DD regimes: ~10.8% of the microbial communities (39 genera) showed daily variation in the LD group and ~9.7% (37 genera) responded similarly in the DD group (Fig. S4). There were genera with identified circadian rhythms shared between the two soil types, with rhizosphere DD samples sharing genera with bulk soil LD (15 genera) and DD (14 genera) samples. There was a smaller number of genera shared by the rhizosphere LD samples, with only two genera shared with bulk soil LD and three genera shared with bulk soil DD. The DD rhizosphere microbial communities were therefore more similar than the LD rhizosphere communities to the bulk soil communities.

Random forest analysis was further used to identify indicator taxa with AM versus PM differences within the LD and DD groups, and was ranked in order of Mean Decrease Accuracy (MDA) index to indicate the contribution of genera to community diel changes. Only two genera with circadian rhythms in the LD group were identified as indicator taxa. In contrast, five genera were identified as indicator taxa in DD; these genera also had a higher MDA value sum (Fig. 3, LD vs DD = 5.38 vs 9.09). Within the bulk soil communities, eight indicator genera with circadian rhythms were identified in the LD regime while nine genera were identified in the DD regime; the contribution of these indicator genera were similar for both treatments (Fig. S4, MDA value, LD vs DD = 14.89 vs 16.01). The number of circadian taxa identified as indicator taxa for each treatment over the two soil types suggests that while the plants influence microbial communities in the dark, microbial circadian clocks might still play a more important role than light-induced diurnal fluctuations in regulating the circadian rhythms of rice rhizosphere microbial communities under a DD regime.

### Meta-community co-occurrence networks

**Fig. 4:**
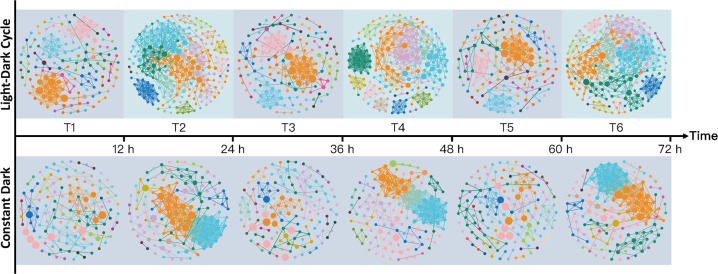
Rhizosphere co-occurrence networks at genus taxonomy level. Nodes represent unique genera; node size is proportional to abundance and node colors indicate modules.

Light exposure significantly changed the topological features of the co-occurrence networks at the genus level (Fig. 4 and Supplementary Table S3). LD daytime networks had more nodes and edges than any dark sample group, and also included a larger proportion of OTUs, higher average degree, normalized closeness centrality and normalized betweenness centrality. Furthermore, LD networks had fewer modules, indicating that light samples had higher niche overlap than dark samples regardless of treatment. When compared to randomly generated networks containing the same node and edge numbers, differences in network topology indicate that the experiment networks were not random (Fig. S5 and Supplementary Table S4).

## Discussion

Rice rhizosphere microbial communities had observable diurnal cycles under both LD and DD treatments, a result that is in contrast to gut microbial communities which have been observed to lose their circadian rhythms when exposed to DD conditions [22]. In addition from the distinctly different environments that soil microbes and gut microbes live in, difference between these two systems may result from RNA-based microbial community analysis being a more sensitive measure for detecting exogenous and endogenous changes than a DNA-based community [51, 52]. Due to the inherent relationships between community composition and environmental factors such as oxygen, pH, and DOC, microbiota co-occurrence patterns varied less over the 3-day observation window in the DD compared to the LD group. The response of rhizosphere microbial communities suggests that there may be entrained rhythms in the LD group [53], whereas the response of bulk soil microbial communities suggests intrinsic rhythms in the DD group. Notably, the patterns of diel divergence between the LD and DD groups were completely different: microbes driven by circadian clocks (as observed in DD results) were masked in the LD treatment by the overall community responses to diel environmental drivers. As a result of this masking, only one taxon was observed with both entrained and endogenous rhythms.

Rice photosynthesis induced an increase in oxygen and DOC during the light period and led to unique microbial assemblies in light versus dark groups, possibly decreasing the contribution of microbial circadian clocks. Oxygen secreted by rice roots during photosynthesis alters the redox and electron supply status of an otherwise anaerobic environment [31, 54], leading to clear patterns in rhizosphere microbial communities [55]. Carbon resources are a well-known driver of microbial assembly, with taxa existing along a spectrum of copiotrophy to oligotrophy based on their adaption to resource availability [56]. Copiotrophs exhibit high growth rates when resource conditions are abundant, while oligotrophs have higher substrate affinities under conditions of low nutrient availability [57]. Our results indicate that the light group had fewer ecological niches and higher niche overlap, and therefore that microorganisms may have temporal complementarity due to changes in niche preferences [58]. This relationship implies that time is a key niche axis that minimizes interspecific competition across the diel cycle [59, 60].

Microbial communities were less stable under light conditions than dark, which is likely a direct response to environmental fluctuations. Light group co-occurrence networks were characterized by properties that indicate low stability, such as higher connectivity and normalized betweenness centrality, and lower modularity [61]. Of these properties, normalized betweenness centrality values were significantly higher in nodes from the light versus dark networks, indicating that the network structures of the light networks depends more on individual taxa and thus potentially have decreased community resilience to environmental stressors [62].

Circadian rhythms are expected to enhance organism fitness by improving their ability to adapt to external influences [63–65]. It is therefore reasonable to infer that entrained rhythms would be less advantageous to organisms living under constant environments [1]. Although it was assumed that organisms might lose their rhythms in the absence of periodicity, circadian rhythms are observed even in organisms that have spent many generations in apedriodic environments [2]. Nevertheless, circadian rhythms are not necessarily beneficial to the organism if the external force is removed or otherwise altered [66–68]. In our study, a majority of genera appeared to lose their rhythms under constant darkness, a pattern also observed in Cyanobacteria [69]. Although such as response suggests an adaptive property of a circadian clock in Cyanobacteria [69], it actually indicates that this adaption is activated by rhythmic environments, an argument that is supported by mathematical modeling of Cyanobacterial fitness under arbitrary light/dark schedules [70]. Furthermore, there is evidence of endogenous redox rhythms driven by oxidative stress and controlled by circadian oscillators which then disappear when measured under DD conditions [71–73]. However, the loss of a particular circadian rhythm does not necessarily mean damage to the circadian system; it is likely that circadian oscillators are functioning normally when involved in other processes, but that they become partially uncoupled from their circadian-related activities [53].

In contrast to studies showing a loss of rhythms under dark conditions, our results show that microbiota without observable circadian rhythms under LD cycles appeared to acquire them when exposed to the DD treatment. The apparent development of circadian rhythms in otherwise non-circadian organisms after entrainement by light cycles is thought to be controlled by circadian clocks [74]. Cave dwelling millipedes, for example, demonstrated a circadian rhythm in locomotive activity after entrainment with a LD cycle [75]. The capacity for circadian entrainment of organisms in otherwise aperiodic environments indicates an intrinsic adaptation value of circadian clocks, possibly due to a requirement to coordinate internal metabolic processes or regulate seasonal breeding [53]. It is also possible that biological clocks exist in steady states ranging from arrhythmic to rhythmic. Therefore, a small fluctuation or stimulus could change an existing arrhythmic state into a rhythmic state or vice versa [1, 76]. Further research is required to identify consistently reliable markers for circadian rhythms in prokaryotes, such as peroxiredoxins, that are more conserved and widespread than *KaiA, KaiB,* and *KaiC* [14, 77]. Moreover, our study only provides a short time scale perspective on microbial and plant–microbe interactions; further research examining the effects of contrasting time scales and other rhythmic factors are required to minimize the impacts of hidden deterministic and stochastic factors.

In summary, our study confirmed that light exposure mediates changes in the circadian rhythms of rice rhizosphere microbial communities, and suggests that light-induced diurnal fluctuations in the rhizosphere are a stronger determinant than microbial circadian clocks in regulating circadian rhythms. Our results reveal a distinct diurnal niche differentiation under different light conditions; microbial communities in light conditions had fewer ecological niches and higher niche overlap than those in dark conditions, but were also less stable. Importantly, as differences in rhizosphere community structure could lead to functional variation [29, 78], our results raise the possibility that rhizosphere microbial community functions could be regulated by above ground light exposure.

## Supplementary information

Supplemental tables and legends of supplemental figures

Supplemental figure 1

Supplemental figure 2

Supplemental figure 3

Supplemental figure 4

Supplemental figure 5
