# Supplementary material for: Light exposure mediates circadian rhythms of rhizosphere microbial communities
Source: ISME J. 2021 Mar 21;15(9):2655–64. doi: 10.1038/s41396-021-00957-3 (PMC8397761; doi:10.1038/s41396-021-00957-3)
Supplement: Supplementary file 1 — Supplemental tables and legends of supplemental figures [file 41396_2021_957_MOESM1_ESM.docx]

**Light exposure mediates circadian rhythms of rhizosphere microbial communities**

Kankan Zhao^1,2 #^, Bin Ma^1,2,3 #*^, Yan Xu^1,2^, Erinne Stirling^1,2,4^, Jianming Xu^1,2*^

^1^ Institute of Soil and Water Resources and Environmental Science, College of Environmental and Resource Sciences, Zhejiang University, Hangzhou 310058, China

^2^ Zhejiang Provincial Key Laboratory of Agricultural Resources and Environment, Zhejiang University, Hangzhou 310058, China

^3^ Hangzhou Global Scientific and Technological Innovation Center，Zhejiang University, Hangzhou 310058, China

^4^ Acid Sulfate Soils Centre, School of Biological Sciences, The University of Adelaide, Adelaide, South Australia, 5005, Australia

^#^ These authors contributed equally.

**^*^** Corresponding author:

Jianming Xu, email: jmxu@zju.edu.cn, tel: +86-13396577916, ORCID: 0000-0002-2954-9764;

Bin Ma, email: bma@zju.edu.cn, tel: +86-13282198979, ORCID: 0000-0003-4807-4992

Running title: Circadian rhythm of microbial community

**Table S1** Time variables (day and night) affecting rhizosphere microbial community composition.

|  | **Constant Dark AM**  **vs**  **Constant Dark PM** | | **Constant Dark AM**  **vs**  **Light-Dark Cycle AM** | | **Constant Dark AM**  **vs**  **Light-Dark Cycle PM** | | **Constant Dark PM**  **vs**  **Light-Dark Cycle AM** | | **Constant Dark PM**  **vs**  **Light-Dark Cycle PM** | | **Light-Dark Cycle AM**  **vs**  **Light-Dark Cycle PM** | |  |
| --- | --- | --- | --- | --- | --- | --- | --- | --- | --- | --- | --- | --- | --- |
| SS | | 0.183 | | 0.112 | | 0.796 | | 0.300 | | 0.772 | | 0.664 | |
| F. Model | | 2.78 | | 1.38 | | 5.11 | | 4.58 | | 5.48 | | 4.24 | |
| R^2^ | | 0.065 | | 0.034 | | 0.113 | | 0.101 | | 0.120 | | 0.096 | |
| p | | 0.014 | | 0.160 | | < 0.001 | | < 0.001 | | < 0.001 | | < 0.001 | |

^a^ SS, sums of squares.

^b^ Variation was based on Weighted Unifrac distances.

^c^ *P* value based on PERMANOVA (999 permutations).

**Table S2** Time variables (day and night) affecting bulk soil microbial community composition.

|  | **Constant Dark AM**  **vs**  **Constant Dark PM** | | **Constant Dark AM**  **vs**  **Light-Dark Cycle AM** | | **Constant Dark AM**  **vs**  **Light-Dark Cycle PM** | | **Constant Dark PM**  **vs**  **Light-Dark Cycle AM** | | **Constant Dark PM**  **vs**  **Light-Dark Cycle PM** | | **Light-Dark Cycle AM**  **vs**  **Light-Dark Cycle PM** | |  |
| --- | --- | --- | --- | --- | --- | --- | --- | --- | --- | --- | --- | --- | --- |
| SS | | 0.066 | | 0.049 | | 0.063 | | 0.069 | | 0.061 | | 0.057 | |
| F. Model | | 1.859 | | 1.958 | | 1.602 | | 2.321 | | 1.275 | | 2.040 | |
| R^2^ | | 0.064 | | 0.065 | | 0.054 | | 0.069 | | 0.045 | | 0.068 | |
| p | | 0.039 | | 0.065 | | 0.047 | | 0.003 | | 0.222 | | 0.018 | |

^a^ SS, sums of squares.

^b^ Variation was based on Weighted Unifrac distances.

^c^ *P* value based on PERMANOVA (999 permutations).

**Table** **S3** Topological features of the co-occurrence networks of sample data.

|  | **Light-Dark Cycle AM** | | |  | **Light-Dark Cycle PM** | | |  | **Constant Dark AM** | | | |  | | **Constant Dark PM** | | |
| --- | --- | --- | --- | --- | --- | --- | --- | --- | --- | --- | --- | --- | --- | --- | --- | --- | --- |
|  | **T1** | **T3** | **T5** |  | **T2** | **T4** | **T6** |  | **T1** | **T3** | **T5** |  | | **T2** | | **T4** | **T6** |
| **Nodes** | 153 | 152 | 144 |  | 240 | 220 | 227 |  | 129 | 137 | 139 |  | | 141 | | 140 | 143 |
| **Edges** | 321 | 338 | 305 |  | 918 | 874 | 858 |  | 167 | 168 | 173 |  | | 518 | | 467 | 519 |
| **Modules** | 36 | 35 | 34 |  | 15 | 15 | 15 |  | 28 | 32 | 32 |  | | 16 | | 18 | 17 |
| **Average degree** | 4.20 | 4.45 | 4.24 |  | 7.65 | 7.95 | 7.56 |  | 2.59 | 2.45 | 2.49 |  | | 7.35 | | 6.67 | 7.26 |
| **Clustering coefficient** | 0.708 | 0.711 | 0.707 |  | 0.513 | 0.645 | 0.475 |  | 0.556 | 0.563 | 0.553 |  | | 0.694 | | 0.670 | 0.694 |
| **Centralization closeness** | 0.002 | 0.002 | 0.003 |  | 0.006 | 0.006 | 0.007 |  | 0.003 | 0.003 | 0.003 |  | | 0.005 | | 0.005 | 0.005 |
| **Centralization betweenness** | 0.007 | 0.008 | 0.009 |  | 0.046 | 0.044 | 0.049 |  | 0.029 | 0.025 | 0.025 |  | | 0.019 | | 0.018 | 0.019 |

**Table S4** Topological features of the co-occurrence networks of random data.

|  | **Light-Dark Cycle AM** | | |  | **Light-Dark Cycle PM** | | |  | **Constant Dark AM** | | | |  | | **Constant Dark PM** | | |
| --- | --- | --- | --- | --- | --- | --- | --- | --- | --- | --- | --- | --- | --- | --- | --- | --- | --- |
|  | **T1** | **T3** | **T5** |  | **T2** | **T4** | **T6** |  | **T1** | **T3** | **T5** |  | | **T2** | | **T4** | **T6** |
| **Nodes** | 153 | 152 | 144 |  | 240 | 220 | 227 |  | 129 | 137 | 139 |  | | 141 | | 140 | 143 |
| **Edges** | 556 | 550 | 528 |  | 1458 | 1158 | 1280 |  | 427 | 451 | 472 |  | | 479 | | 446 | 499 |
| **Modules** | 8 | 7 | 7 |  | 7 | 8 | 7 |  | 7 | 8 | 8 |  | | 8 | | 8 | 7 |
| **Average degree** | 7.27 | 7.24 | 7.33 |  | 12.15 | 10.53 | 11.28 |  | 6.62 | 6.76 | 6.79 |  | | 6.79 | | 6.99 | 6.98 |
| **Clustering coefficient** | 0.051 | 0.051 | 0.046 |  | 0.055 | 0.054 | 0.049 |  | 0.050 | 0.045 | 0.054 |  | | 0.044 | | 0.057 | 0.041 |
| **Centralization closeness** | 0.099 | 0.126 | 0.110 |  | 0.112 | 0.130 | 0.130 |  | 0.118 | 0.124 | 0.111 |  | | 0.117 | | 0.152 | 0.125 |
| **Centralization betweenness** | 0.027 | 0.027 | 0.030 |  | 0.013 | 0.020 | 0.018 |  | 0.036 | 0.035 | 0.031 |  | | 0.032 | | 0.050 | 0.036 |

**Fig. S1** Composition of constant dark and light-dark cycle treatments communities at phylum (a), order (b) and genus (c) level.

**Fig.S2** Principal coordinate analysis (PCoA) of Weighted Unifrac dissimilarities (n = 7 for each time point).

**Fig. S3** Alpha diversity based on Shannon (a) and Chao1 (b) indices and 16S rRNA gene copy numbers (c) in all four bulk soil groups (n = 15 for each group). (d) Principal coordinate analysis (PCoA) of Weighted Unifrac dissimilarities.

**Fig. S4** Treatment dynamics of bulk soil microbiota with circadian rhythms (left) and the top 40 indicator taxa associated with the AM and PM measurements (right) in LD (a) and DD (b). In the left-side panel, bubble size indicates normalized abundance of a genus along six time points. Population of each time point was compared to the maximum population of each genera and normalized to a value between 0 to 1. Green bubbles represent taxa with circadian rhythms in DD rhizosphere samples; yellow bubbles represent taxa with circadian rhythms in both LD and DD rhizosphere samples; gray bubbles represent taxa with circadian rhythms in bulk soil only; no taxon with circadian rhythms in LD rhizosphere samples but not DD rhizosphere samples was also present in bulk soil. Polylines connect the same member occurrence in the left- and right- side panel.

**Fig. S5** Random networks with same nodes number for each timepoint. Node colors indicate modules.
