# Supplementary figures and images for: Light exposure mediates circadian rhythms of rhizosphere microbial communities
Source: ISME J. 2021 Mar 21;15(9):2655–64. doi: 10.1038/s41396-021-00957-3 (PMC8397761; doi:10.1038/s41396-021-00957-3)

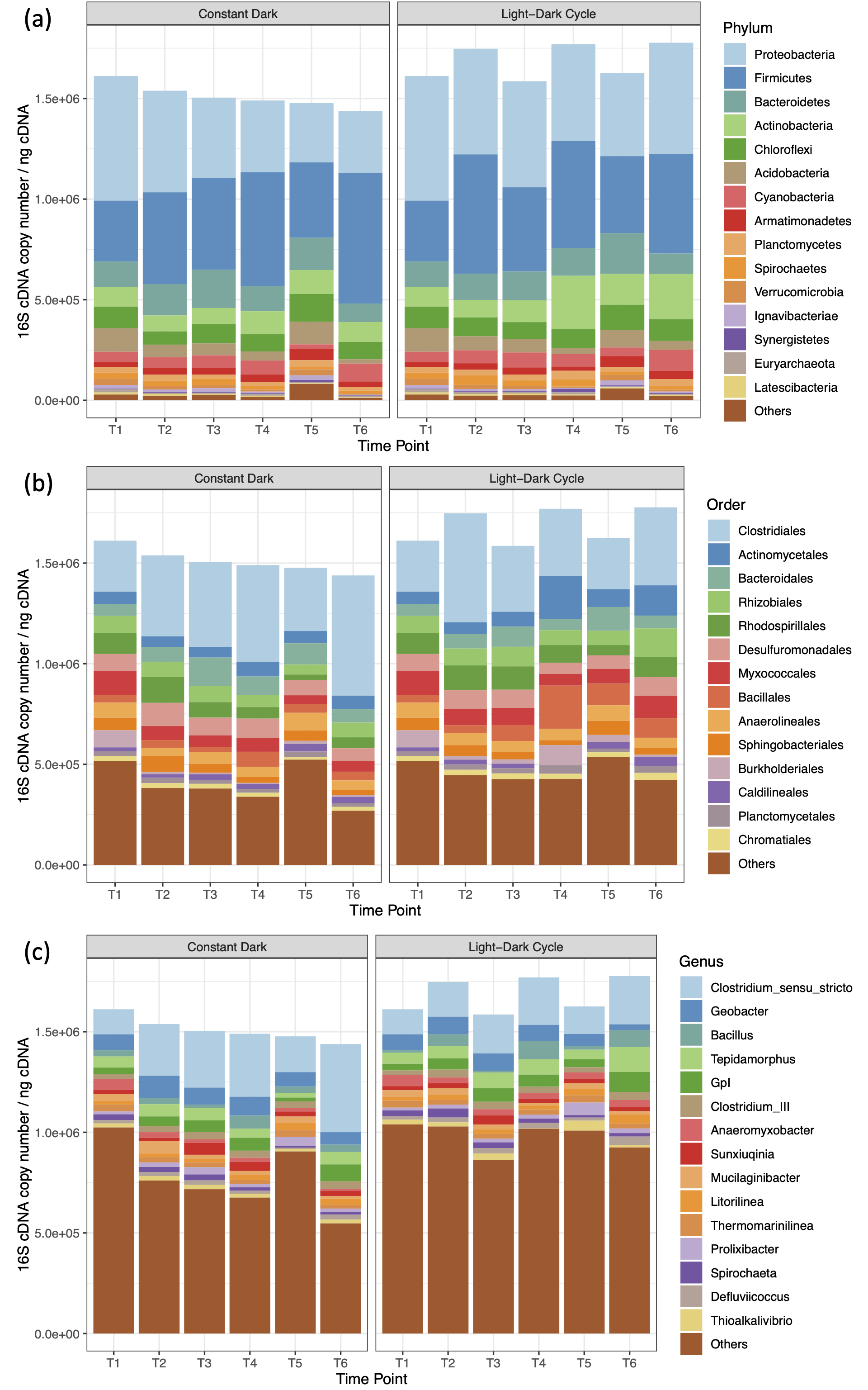

Supplement: Supplementary file 2 — Supplemental figure 1 [file 41396_2021_957_MOESM2_ESM.tif]

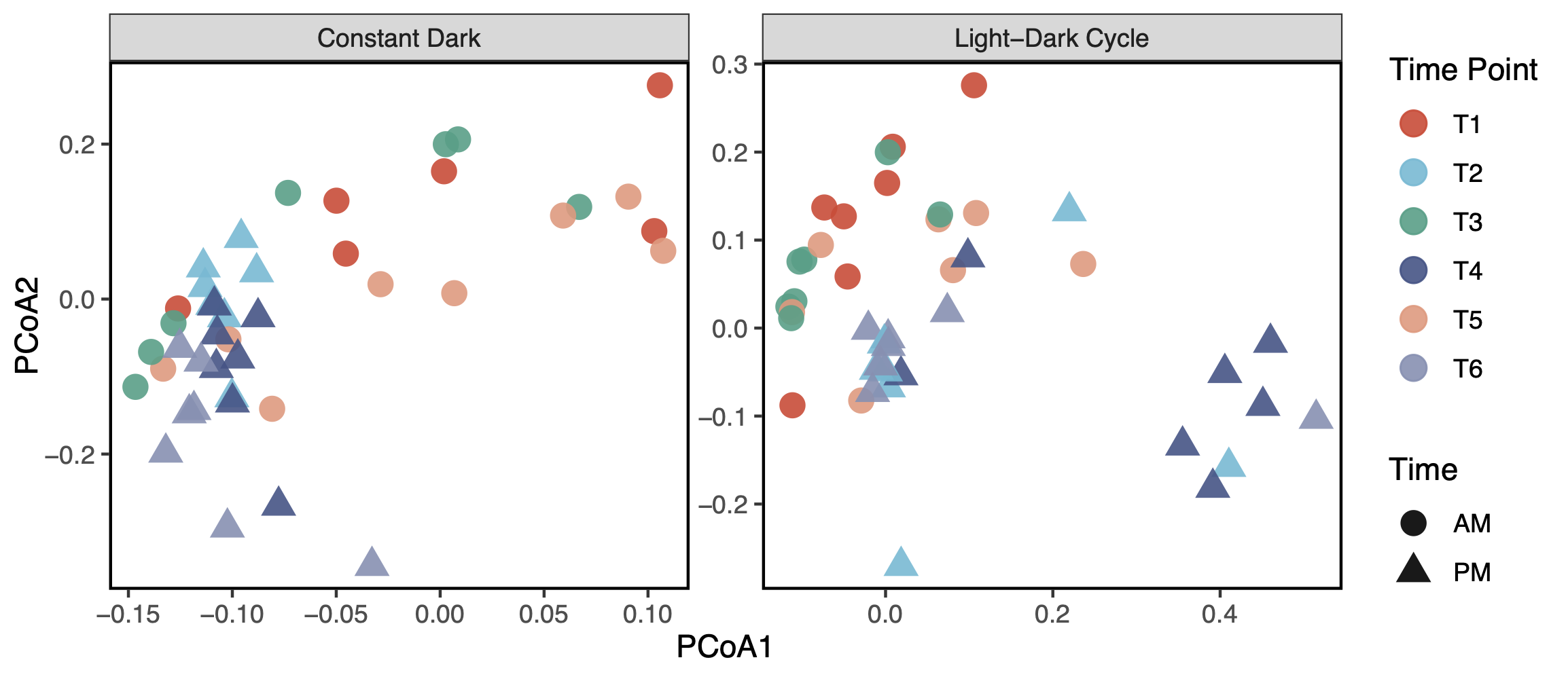

Supplement: Supplementary file 3 — Supplemental figure 2 [file 41396_2021_957_MOESM3_ESM.tif]

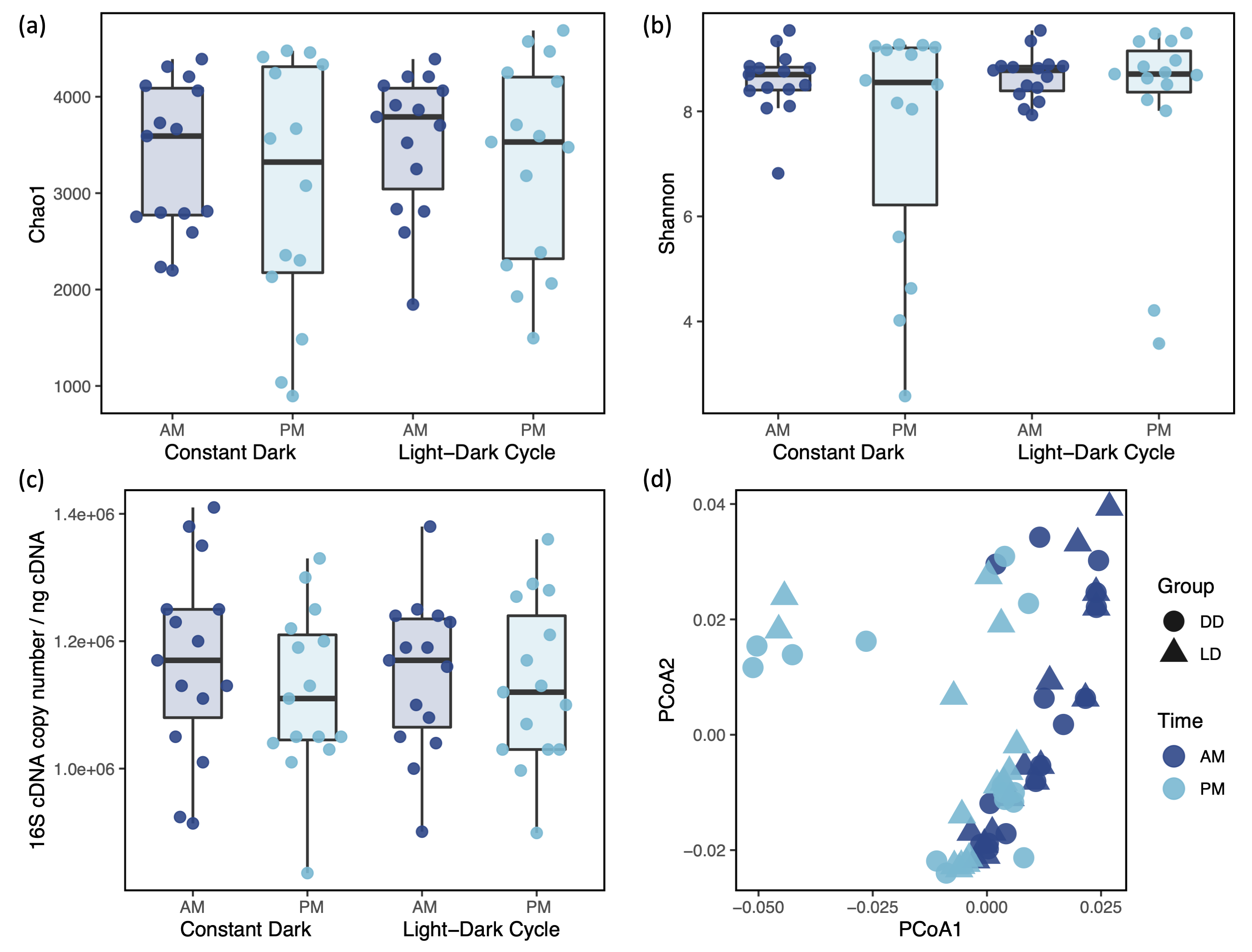

Supplement: Supplementary file 4 — Supplemental figure 3 [file 41396_2021_957_MOESM4_ESM.tif]

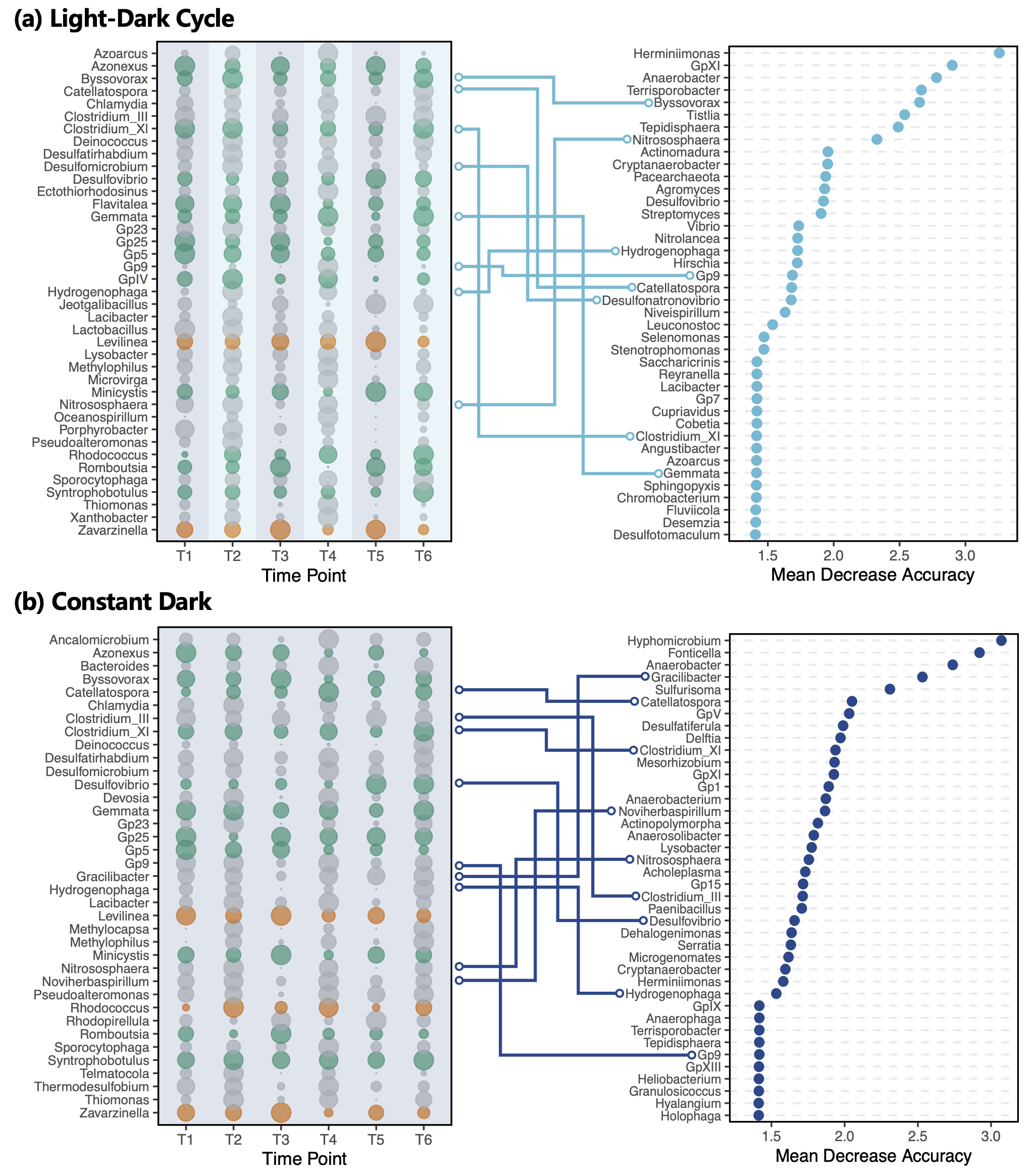

Supplement: Supplementary file 5 — Supplemental figure 4 [file 41396_2021_957_MOESM5_ESM.tif]

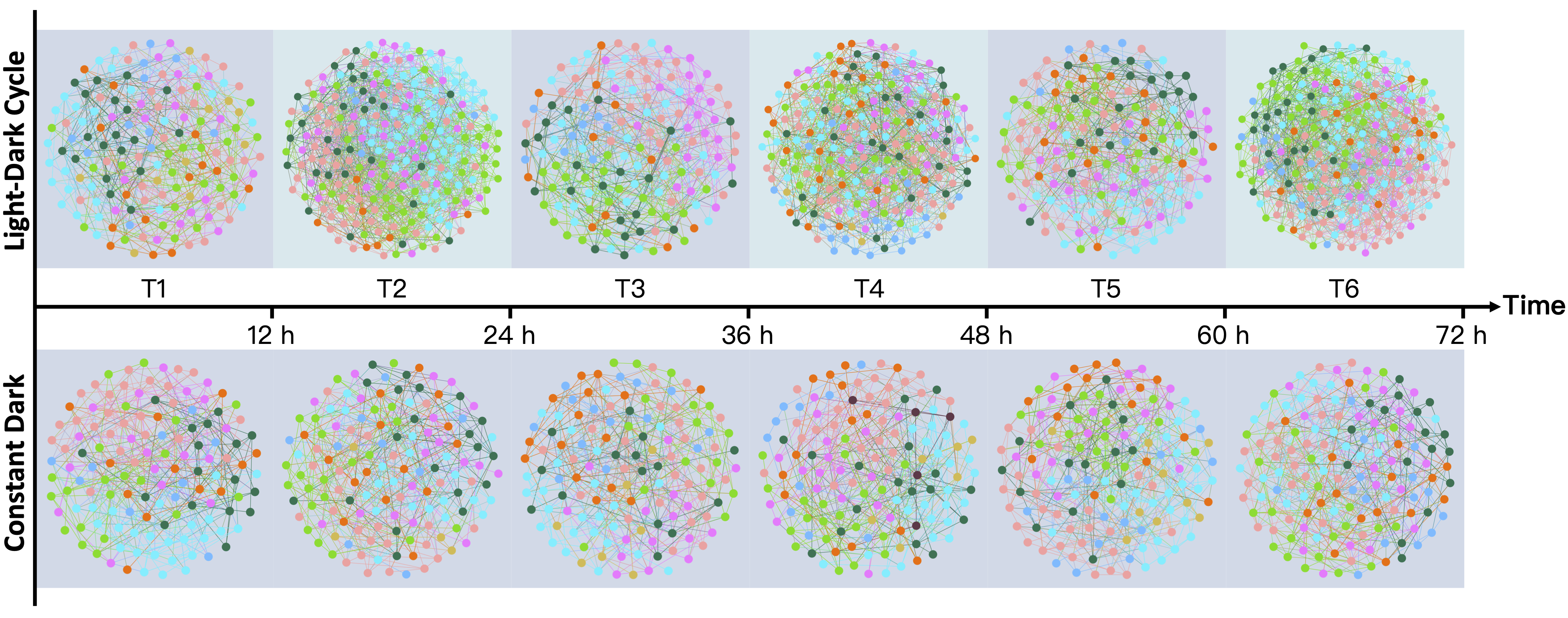

Supplement: Supplementary file 6 — Supplemental figure 5 [file 41396_2021_957_MOESM6_ESM.tif]
